# ﻿*Cardaminetangutorum* O.E.Schulz (Brassicaceae), a new synonym of *Cardaminemacrophylla* Willd

**DOI:** 10.3897/phytokeys.255.146179

**Published:** 2025-04-17

**Authors:** Jia-lu Li, Yi He, Quan-ru Liu

**Affiliations:** 1 Key Laboratory of Biodiversity Science and Ecological Engineering, Ministry of Education, College of Life Sciences, Beijing Normal University, Beijing 100875, China Beijing Normal University Beijing China

**Keywords:** *Cardamine*, China, morphology, phylogenetics, taxonomy

## Abstract

*Cardaminemacrophylla* Willd. and *Cardaminetangutorum* O.E.Schulz are both widely distributed plants of the genus *Cardamine* L. in East Asia, and they are regarded as the same species in *Flora of China*. In this study, through literature and specimen research, morphological measurement and phylogenetic analyses, the results show that the traditional distinguishing characters cannot distinguish the two species. *Cardaminetangutorum* O.E.Schulz inserted into the branch of *Cardaminemacrophylla* Willd. in the molecular phylogenetic tree. Therefore, based on the results of this study, *Cardaminetangutorum* O.E.Schulz was treated as the synonym of *Cardaminemacrophylla* Willd.

## ﻿Introduction

The genus *Cardamine* L. belongs to the tribe Cardamineae within the Brassicaceae family. It is a cosmopolitan group with approximately 280 species distributed worldwide (Marhold et al. 2021). As one of the global centers of diversity, *Flora of China* records 48 species of *Cardamine* L. in China, 24 of which are endemic. New species and distribution records of *Cardamine* L. in China continue to be reported (Al-Shehbaz 2015a, 2015b; Diao et al. 2023)(Marhold et al. 2007; Al-Shehbaz and Boufford 2008; Chen et al. 2011; An et al. 2016; Marhold et al. 2016; Šlenker et al. 2018; Wu et al. 2021; Guo et al. 2024; Li et al. 2024). To date, there are 61 *Cardamine* L. species in China, 31 of which are endemic.

In 1753, Linnaeus published the genus *Cardamine* L. in Species Plantarum, which included 15 plant species. The type species of the genus is *Cardaminepratensis* L. Additionally, Linnaeus also established the genus *Dentaria* L. (Linnaeus 1753). In 1769, Crantz merged *Dentaria* L. into *Cardamine* L. (Crantz 1769). O. E. Schulz classified *Cardamine* L. into 13 sections based on the presence or absence of scales on the rhizome surface, ovule number, funiculus morphology, and the relative position of cotyledons and radicles (Schulz 1903)(Schulz 1931, 1936). This system was adopted by major floras such as FRPS and Flora of the USSR (Komarov 1939; Zhou. et al. 1987). In FRPS, Chinese *Cardamine* L. species were divided into seven sections based on Schulz’s classification system, with most species assigned to the section Cardamine L.

Al-Shehbaz proposed the concept of a broadly circumscribed *Cardamine* L., defining the genus as a cruciferous group characterized by “flattened and winged seeds, and dehiscent siliques that split elastically through torsion.” This treatment incorporates *Dentaria* into the expanded *Cardamines. l.* and abolishes infrageneric classifications (Al-Shehbaz 1988, 2000, 2015c) Al-Shehbaz and Guang 1998; Zhou et al. 2001). These taxonomic revisions have subsequently received support from molecular phylogenetic evidence (Franzke et al. 1998; Sweeney and Price 2000). Major floristic works, including Flora of China and Flora of Pan-Himalaya, have adopted Al-Shehbaz’s taxonomic treatment of *Cardamine* L. (Zhou et al. 2001; Al-Shehbaz 2015c).

During the nomenclatural verification and taxonomic studies of *Cardamine* L. species in China, we found that the identification of specimens for the widely distributed *Cardaminemacrophylla* and *Cardaminetangutorum* in East Asia was inconsistent, leading to controversies in their species delimitation. Flora of China suggested that these two taxa might represent the same species, requiring further verification (Zhou et al. 2001).

*Cardaminemacrophylla* Willd. was first described by Willdenow, based on specimens collected from the Taz River estuary in the Mangazeya region of northern Siberia, USSR (as shown in Fig. 2). The distribution of *Cardaminemacrophylla* spans a wide latitudinal range, occurring in Siberia, the Qinling Mountains, and the Hengduan Mountains. Due to its significant morphological variation, numerous subspecies and varieties have been described under this species. In 1825, D. Don described *Cardaminepolyphylla* D. Don (Don and Hamilton 1825). In 1872, several varieties of *Cardaminemacrophylla* were published in Flora of British India: Cardaminemacrophyllavar.dentariifolia Hook. f.& T. Anderson, Cardaminemacrophyllavar.lobata Hook. f.& T. Anderson, and Cardaminemacrophyllavar.sikkimensis Hook. f.& T. Anderson. Additionally, the nomen nudum *Cardaminefoliosa* Wall. was treated as Cardaminemacrophyllavar.foliosa Hook. f.& T. Anderson (Wallich and East 1828; Hooker 1875). In Schulz’s 1903 system, *Cardaminemacrophylla* was placed in the *Macrophyllum* section (Schulz 1903).

In 1980, Tai Yien Cheo and colleagues treated *Cardaminepolyphylla* D. Don as a variety of *Cardaminemacrophylla* Willd., naming it CardaminemacrophyllaWilld.var.polyphylla (D. Don) T. Y. Cheo et Fang. They also described a new variety, CardaminemacrophyllaWilld.var.diplodonta T. Y. Cheo, based on variations in leaf margin serration and the number of cauline leaves (Cheo et al. 1980). In the FRPS, three varieties of *C.macrophylla* were recognized: C.macrophyllaWilld.var.polyphylla (D. Don) T. Y. Cheo et Fang, C.macrophyllaWilld.var.diplodonta T. Y. Cheo, and Cardaminemacrophyllavar.crenata Trautv. (Trautvetter 1887). However, the 2001 edition of Flora of China synonymized these varieties under *C.macrophylla*. Additionally, it synonymized *Dentariasino-manshurica* Kitag (Kitagawa 1937) and *Cardamineurbaniana* O.E. Schulz (Schulz 1903) with *C.macrophylla* (Zhou et al. 2001).

**Figure 1.**
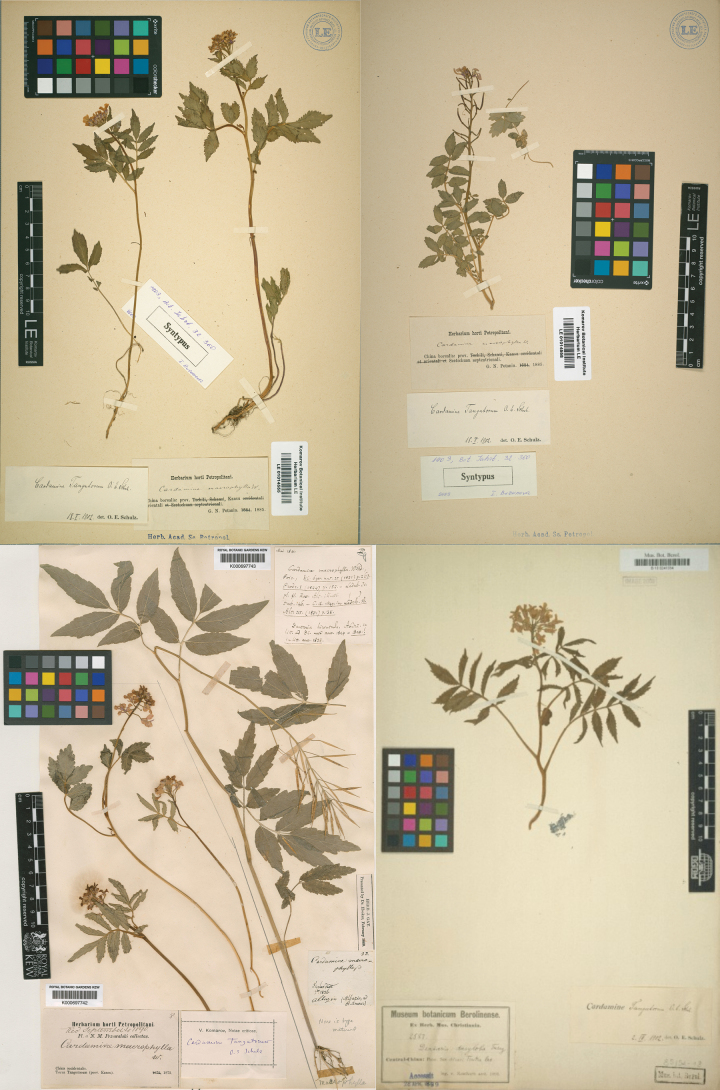
Syntype specimens of *Cardaminetangutorum* including 1880 N. M. Przewalski #s.n. (top left), 1885 G. N. Potanin #s.n. (top right), 1879 N. M. Przewalski #s.n. (bottom left), and Rosthorn #2583 (bottom right).

*Cardaminetangutorum* O.E. Schulz was described by Schulz in 1903. According to the protologue, this species is distinguished by its purplish-red, prominently veined sepals with translucent narrow margins and its triangularly swollen ovule stalks measuring 0.5–4.0 mm in length. Schulz cited multiple specimens as syntypes for this species: Gansu 1872 *N. M. Przewalski #s.n.*,1879 *N. M. Przewalski #s.n.*,1880 *N. M. Przewalski #s.n.*; *Gansu* 1885 *G. N. Potanin #s.n.*; *Shaanxi*, Miaowang Mountain 1899 *J. Giraldi No. 3379 No. 3378* ;Shaanxi 1884 *Potanin #s.n.*; Beijing Xiaowutai Mountain 1879 *Möllendorff #s.n.*; Sichuan 1894 *Rosthorn No. 2583* (see Fig. 1). In Schulz’s classification system for *Cardamine* L., *C.tangutorum* was placed in the section Dentaria (Schulz 1903). Both the FRPS and Flora of China recognized *C.tangutorum*. However, the editors of Flora of China noted the morphological similarity between *C.tangutorum* and *C.macrophylla*, suggesting that they might represent the same species (Zhou. et al. 1987; Zhou et al. 2001), with a common species distributed in subalpine and alpine zones in the Himalayas, North China, Northeast China, Central China, and Hengduan Mountains.

**Figure 2.**
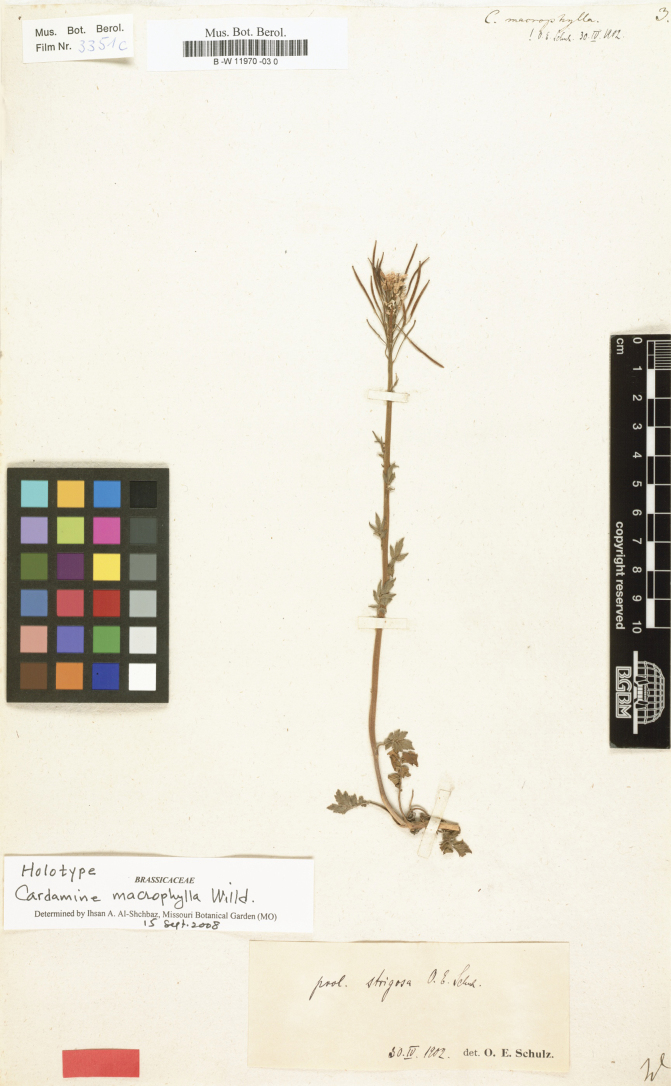
Holotype specimen of *Cardaminemacrophylla*.

According to records from Flora Reipublicae Popularis Sinicae, Flora of China, and Flora of Pan-Himalaya, the morphological distinctions between *Cardaminemacrophylla* and *C.tangutorum* are primarily as follows: *C.macrophylla* exhibits a conspicuously thickened rhizome lacking scale-like appendages, a plant height of (20–) 30–95 (–115) cm, 3–12 cauline leaves scattered along the stem, and uppermost leaflets of the cauline leaves decurrent into winged extensions. *C.tangutorum* is characterized by a slender, whip-like rhizome with scale-like appendages, a plant height of (8–) 15–30 (–40) cm, 1–3 cauline leaves clustered at the upper stem, and comparatively narrower and smaller cauline leaves with fewer lateral leaflets and non-decurrent uppermost leaflets.

Based on the above understanding, we conducted morphological and molecular phylogenetic studies, supported by a thorough review of the literature and examination of specimens, to clarify the taxonomic status of these two species.

## ﻿Method

### ﻿Sampling and morphological analyses

Specimens with complete rhizoid and multiple copies were selected from PE, BNU, KUN and other herbaria, as well as the digital HD and other type specimen photos on the website of LE Herbarium. Specimens’ collection are shown in Table 1. A total of 46 population specimens were collected for morphological analyses. Re-identified and grouped according to the retrieval characters described above. Ten traits were selected according to FRPS, Flora of China, and protologue, and measured by ImageJ (Schindelin et al. 2012), and two ratios were calculated. The characters and coding are listed in Table 2. PCA was performed by Past 4.13 after standardization by IBM SPSS Statistics (v.27) (Hammer et al. 2022; IBMCorp 2020).

**Table 1.** Taxa and sample sites, bold specimen representation was used for molecular phylogenetic analysis.

Taxa and sample sites
***C.tangutorum* O.E. Schulz**
China Sichuan, Zhongsu Team, *7165*
China Sichuan: Wenchuan, K.Y.Lang ,*L.Q.Li, Y. Fei, 1136*
China Sichuan: Aba, K.Y.Lang ,*L.Q.Li, Y. Fei, 2048*
China Qinghai: *K.M.Liou, 6185*
China Qinghai, Z.H.Zhang etc, *4484*
China Gansu, *T.P.Wang 7275*
China Gansu, *T.P.Wang 6979*
China Gansu, *Tsi-Tang Li198*
China Hebei: Tuoliang, *TL006*
China Hebei: Tuoliang, *TL008*
**China Hebei: Tuoliang, *TL023***
**China Hebei: Tuoliang, TL007**
China Beijing: Donglingshan, *C. Wang, 50611006*
China: Eastern Gansu, *G. H. Potani, 1885*
China Gansu: Tanggulashan, *N. M. Przewalski, 1872*
China Hebei: Xiaowutaishan, *62079 CC. W. Wang*
China Gansu: 1879, *N. M. Przewalski #s.n.* (syntype of *C.tangutorum*)
China Gansu: 1880, *N. M. Przewalski #s.n.* (syntype of *C.tangutorum*)
***C.macrophylla* Willd.**
China Chongqing, Chengkou, G212 Road, *zhang698*
China Sichuan, Aba, *zhang801*
China Sichuan: Bazhong, *GSL2015050209*
**China Hunan, longshan, Bamianshan, *zhang667***
**China Hubei, Yanzi Zhen, *zhang676***
China Yunnan, *T.T.yu 9780*
China Sichuan, *S.X, Yu, Y.T.Hou, X.X.Zhang, Y.M.Zhao, 4812*
China Sichuan, *K.Y.Lang, L.Q.Li, Y.Li, 1917*
China Sichuan: Batang, *K.Y.Lang, L.Q.Li, Y.Li, 2471*
China Sichuan: Nanping, *K.Y.Lang, L.Q.Li, Y.Li, 1608*
China Hebei: Xilingshan, *J.X.Duan, 239*
China Shanxi: Nuanshuihe, *Shanxi Investigation Team, 530*
China Shanxi: Shiziping, *Shanxi Investigation Team, 592*
China Shanxi: Yuwu, *J.M.Liu, 1662*
China Henan: Neixiang, *D.E.Boufford,C.Y.Xi,T.S.Ying etc.26303*
China Hubei: Shennongjia, *Hubei Shennongjia Planting Research Team, 10006*
China Anhui: Yuexi, *X.L.Liu, 492*
China Hunan: Sangzhi, *B. Zhang & X. Xiang, 090425022*
China Sichuan: Kangding, *Chuanxi Team, K.J.Guan, W.C.Wang etc,772*
China Sichuan: Kangding, *Chuanxi Team, K.J.Guan, W.C.Wang etc,387*
China Sichuan: Kangding, *Y.T.Zhang & K.Y.Lang, 37*
China Sichuan: Ganzi, *Xizang Team, 73-03*
China Sichuan: Ganzi, *Y.T.Zhang & K.Y.Lang, 121*
China Sichuan, E.H.Li,*Y.F.Han, J.G.Liao, Y. Hu, H82-335*
China Sichuan: Muli, *Qingzang Team, 13042*
China Sichuan: Xiangcheng, *Qingzang Team & Hengduanshan Team, 003712*
China Sichuan: Yanyuan, *Qingzang Team, 12344*
China Sichuan: Maerkang, *X. Li, 70477*
China Sichuan: Jiulong, *Z.X.Tang, X.W.Tian, Q.G.Sun, 245*

**Table 2.** List of morphological characters and their acronyms used in analyses.

Acronym	Description of character
Scaly*	Presence of scales on rhizome (with 1/without 0)
RW	Rhizome diameter
CW	Mid-stem diameter
H*	Height of stem
LH*	Height of the location of lowest stem leaves
No. SL*	Number of stem leaves
No. MLL*	Number of lateral leaflets on mid-stem leaves
LTL*	Length of the terminal leaflet on mid-stem leaves
WTL	Width of the terminal leaflet on mid-stem leaves
DL*	Length of the uppermost leaflet’s lower edge on mid-stem leaves
Ratio	
LH/H*,LTL/WTL*,RW/CW*	

### ﻿Phylogenetic analyses

Fresh plant leaves were collected in the field and quickly dried with silica gel. Plant samples were sent to Beijing Novogene Corporation for quality testing and re-sequencing. The sequencing platform, Illumina HiSeq X Ten and BGI, was used to generate approximately 6 GB of data for each sample. The chloroplast genome was assembled from the clean data using Get Organelle (Jin et al. 2020). Plastid Genome Annotator (PGA) was used to annotate the chloroplast genome with *Amborellatrichopoda* Baill. from software as references (Qu et al. 2019). Then, 26 plastid genome sequences were downloaded from NCBI (Table 7), including 23 species of *Cardamine* L.and 2 species, *Rorippasylvestris* (L.) Besser, *Rorippaindica* (L.) Hiern as outgroup. The annotated sequences were imported into PhyloSuite (Zhang et al. 2020), the Mafft module was used for sequence alignment (Katoh et al. 2019), and the ModelFinder module was used to calculate the nucleotide substitution model for the aligned sequences. The maximum likelihood (ML) tree was constructed using IQ-TREE (Minh et al. 2020), with the nucleotide substitution model set to GTR+R3+F and a standard bootstrap value of 1000.

### ﻿Flow cytometric measurements and estimation of DNA ploidy levels

Methods referring to Marhold et al. (2010) and Kobrlová and Hroneš (2019) measured the nuclear DNA content using flow cytometry. Inferred the DNA ploidy levels within the studied populations based on *Cardamine* L.species with known ploidy. The relative nuclear DNA content was determined using PI, a DNA intercalating fluorescent dye, with arbitrary units (a.u.) as the unit of measurement. The buffer solution used was LB01. Dehydrated leaves, preserved by drying at 40 °C for 18–24 months, were used for the determination of chromosome ploidy. The sample sources and voucher specimens are presented in Table 3. In a pre-cooled culture dish, 1–2 mL of LB01 buffer solution and 2 cm^2^ of dry leaves were added. After rapid chopping, the mixture was filtered through a 400-mesh gauze, centrifuged at 4 °C, 3000 rpm for 10 minutes, and the supernatant was discarded. The pellet was re-suspended in 600 μL of LB01 buffer solution, followed by the addition of 100 μL of PI solution (50 μg/mL), and stained in the dark for 15 minutes. Ploidy level of the stained cell suspension was determined by flow cytometry (ACEA NovoCyte 3130). Using 488 nm blue light excitation, 10,000 cells were collected at a time. The other samples were determined under the same voltage bar using C. scutata (2n = 4x = 32) as the reference for tetraploid.

**Table 3.** List of taxa and sample sites used in flow cytometric measurements.

Taxa	Voucher information
*C.tangutorum*	BNU2023WLH077
	BNU2022xz
	BNU2023ZJK26
	BNU2022HLG 002
*C.macrophylla*	BNU2022YN070
	BNU2022YN004
	BNU2022mcs006
	BNU2022em002

## ﻿Result

### ﻿Principal component analysis results

Using SPSS software, a cluster analysis was conducted on the standardized data matrix, resulting in a character correlation matrix as shown in Table 4. The cluster analysis results indicate that none of the Pearson correlation coefficients between each pair of characters reached 0.8. The highest correlation was between the length of the apical leaflet of the stem leaves and the downwards extension length of the first pair of lateral leaflets, with a coefficient of 0.753. Thus, no significant correlations were observed among other traits, allowing all traits to be retained for subsequent multivariate principal component analysis.

**Table 4.** Correlation of morphological characters between *C.macrophylla* and *C.tangutorum*.

	H	LHH	No. SL	No. MLL	LTL	LTL/WTL	DL	RW/CW	SCALY
H	–	-0.16	0.209	0.402	0.597	0.209	0.509	0.295	0.037
LHH		–	-0.653	-0.136	-0.161	0	-0.08	-0.05	0.041
No. SL			–	0.114	0.162	0.171	0.201	0.049	-0.052
No. MLL				–	0.298	0.093	0.312	0.16	0.002
LTL					–	0.267	0.753	0.431	0.271
LTL/WTL						–	0.325	0.102	-0.03
DL							–	0.37	0.045
RW/CW								–	0.119
SCALY									–

**Figure 3.**
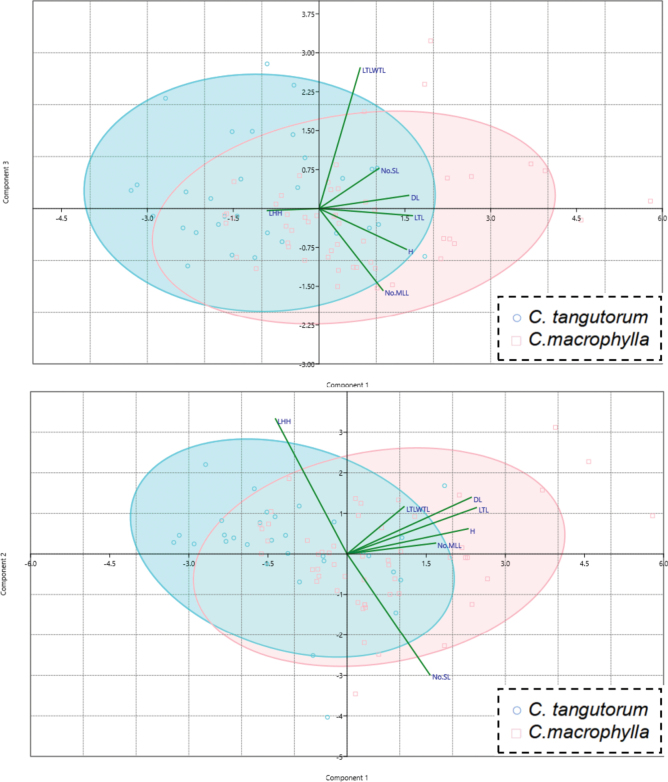
Ordination diagrams of principal component analyses, Grouping reference specimen identification.

Principal component analysis (PCA) was performed on the standardized data matrix using PAST software, and the contribution values of each principal component (PC) are shown in Table 5. The cumulative contribution of the first four principal components was 77.658%, indicating successful dimensionality reduction, making PCA suitable for this study. Based on previously identified results, the data was grouped and incorporated into the standardized data matrix, as shown in Fig. 3.

**Table 5.** Contribution Values of Each Principal Component.

Principal component (PC)	Eigenvalue	Proportion (%)	Cumulative (%)
PC1	2.938	36.615	36.615
PC2	1.511	18.828	55.443
PC3	0.956	11.919	67.362
PC4	0.826	10.296	77.658
PC5	0.601	7.493	85.151
PC6	0.475	5.917	91.067
PC7	0.351	4.368	95.435
PC8	0.244	3.0354	98.471
PC9	0.123	1.5291	100.000

**Figure 4.**
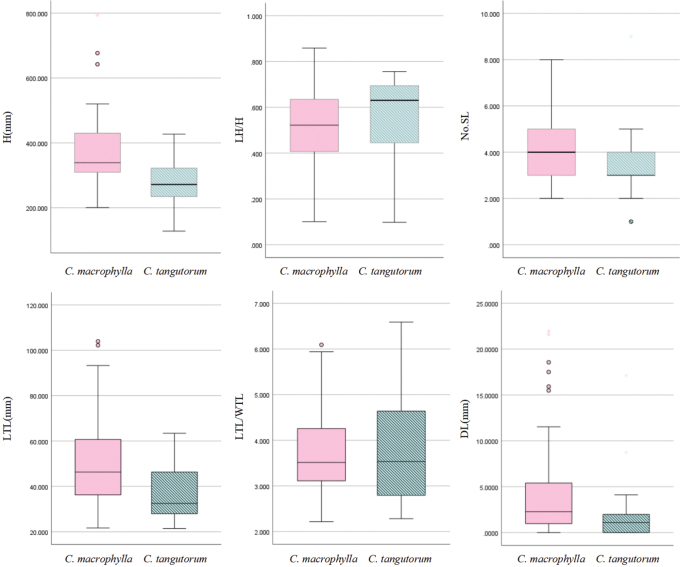
Box Plots of Traits with High Principal Component Contributions.

Based on the results of the PCA, the distribution ranges of *C.macrophylla* and *C.tangutorum* along the PC3 and PC2 axes are essentially identical. Along the PC1 axis, the two species exhibit a clear continuous transitional distribution, with significant overlap in their primary distribution ranges and their core distribution areas also show substantial overlap. In PC1, characteristics with larger contributions include the length of the downwards extension of the uppermost lateral leaflets of the stem leaves, the length of the apical leaflets of the stem leaves, and plant height. In PC2, the characteristic with the largest contribution is the position of leaf attachment. For PC3, the characteristics with the highest contributions are the leaf length-to-width ratio and the number of lateral leaflets on the stem leaves. Box plots of these high-contributing traits, as shown in Fig. 4, indicate that the distribution ranges of all these high-contributing characteristics also demonstrate significant overlap.

### ﻿Molecular phylogenetic study

**Figure 5.**
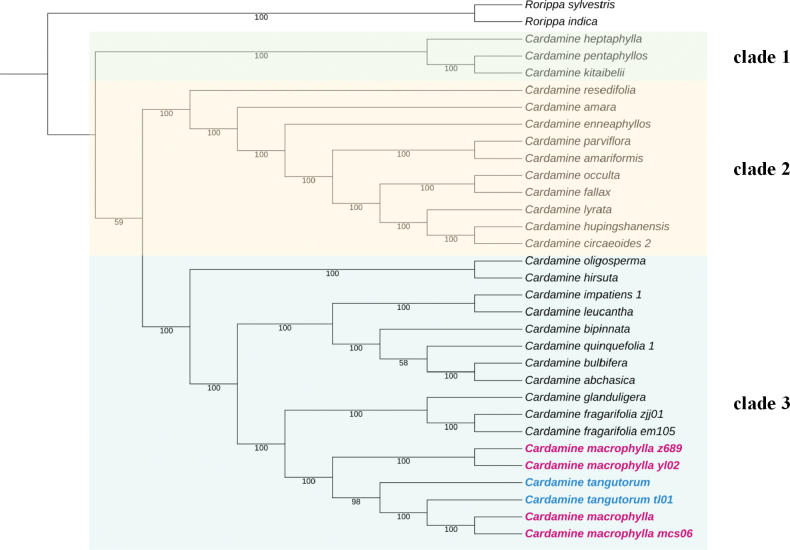
The strict consensus tree resulted from IQ-TREE analysis using plastid genome sequence. Bootstrap values (BS) are shown under branches. Red marking *C.macrophylla*, blue marking *C.tangutorum*.

The chloroplast genome tree is illustrated in Fig. 5, with bootstrap support (BS) values indicated below the branches. The *Cardamine* L. species selected for this study form a well-supported monophyletic group, divided into three distinct clades. *C.tangutorum* is nested within the monophyletic group formed by *C.macrophylla*, both clustering together in clade 3. Besides *C.macrophylla* and *C.tangutorum*, morphologically similar species such as *C.leucantha* and *C.fragarifolia* were included in constructing the phylogenetic tree.*C.leucantha* clusters with *C.impatiens* at the base of clade 3. *C.leucantha* forms a monophyletic group with *C.glanduligera*, positioned within clade 3.

### ﻿Flow cytometric measurements and estimations of relative DNA content

**Figure 6.**
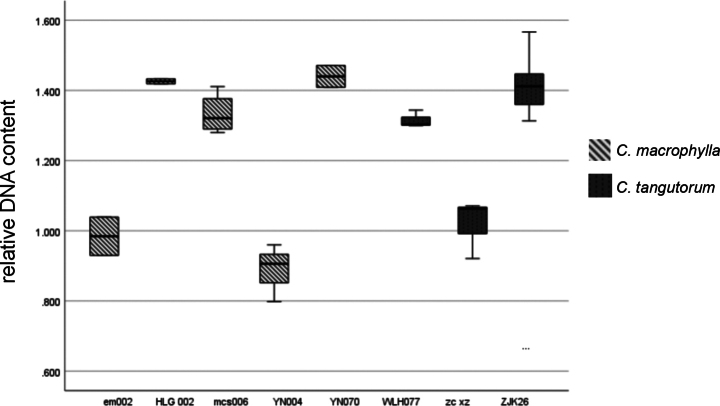
Relative genome sizes obtained for *C.macrophylla*, *C.tangutorum*.

The relative DNA content between different populations of *C.macrophylla* and *C.tangutorum* was calculated using 12-to 24-month-old specimens. The results showed that for all the populations measured in this study, the coefficient of variation ranged from 0.49% to 6.89%, as shown in the Table 6, indicating that the results were credible and that the relative DNA content of *C.macrophylla* and *C.tangutorum* was stable within the populations. The relative DNA content of *C.macrophylla* and *C.tangutorum* is shown below; the relative DNA content of *C.macrophylla* and *C.tangutorum* varies greatly among populations, but the distribution range at the species level is basically the same (shown in Fig. 6).

**Table 6.** Relative genome sizes obtained for *C.macrophylla, C.tangutorum*.

Taxa	Voucher information	Relative genome size in a.u. (arbitrary units)	Variation (%)
*C.tangutorum*	BNU2023WLH077	1.316	1.89%
*C.tangutorum*	BNU2022xz	1.018	6.49%
*C.tangutorum*	BNU2023ZJK26	1.415	6.13%
*C.macrophylla*	BNU2022YN070	1.440	3.10%
*C.macrophylla*	BNU2022YN004	0.888	6.02%
*C.macrophylla*	BNU2022mcs006	1.333	4.33%
*C.macrophylla*	BNU2022em002	0.985	5.45%
*C.tangutorum*	BNU2022HLG 002	1.426	0.49%

**Table 7.** Taxa in phylogenetic analyses from GenBank.

GenBank accession numbers	Species name
NC026446	*C.resedifolia*
MT136871	*C.quinquefolia*
MK637691	*C.pentaphyllos*
NC036964	*C.parviflora*
NC036963	*C.oligosperma*
MZ043777	*C.occulta*
MF405340	*C.macrophylla*
MZ846206	*C.lyrata*
MK637684	*C.kitaibelii*
NC026445	*C.impatiens*
ON322745	*C.hupingshanensis*
MK637681	*C.hirsuta*
MN651504	*C.heptaphylla*
MK637680	*C.glanduligera*
MZ043778	*C.fallax*
NC049605	*C.enneaphyllos*
OL634846	*C.circaeoides*
NC049603	*C.bulbifera*
MN651509	*C.bipinnata*
MZ043776	*C.amariformis*
NC036962	*C.amara*
NC060863	*C.abchasica*
KJ136821	*C.impatiens*
NC069649	*Rorippasylvestris*
NC065833	*Rorippaindica*

## ﻿Discussion

### ﻿Taxonomic status of *C.tangutorum*

Through morphological analysis of 46 populations, it was found that the distinguishing features between *C.macrophylla* and *C.tangutorum* significantly overlap. The decurrence in leaflets of *C.macrophylla* and *C.tangutorum* significantly overlaps in their distribution ranges. Generally, the decurrence in leaflets of *C.macrophylla* tends to be longer. However, some populations, such as *Qingzang Expedition 12344* and *T.T.yu 9780*, exhibit nearly no decurrence in leaflets. Other related species within the genus, such as *C.lyrata* and *C.occulta*, exhibit continuous variability in decurrence in leaflets, making this trait unsuitable as a basis for species differentiation. The position of stem leaves is also a significant distinguishing feature in FRPS (Zhou. et al. 1987), but as shown in the boxplot, the lower stem leaf positions of both *C.macrophylla* and *C.tangutorum* are essentially the same, located in the upper part of the plant, cannot be used for distinguishing.

Previously, when *C.tangutorum* was described, Schulz cited many collections as syntype specimens. Comparing the syntype specimens of *C.tangutorum* with the holotype specimens of *C.macrophylla*, decurrence in leaflets cannot be observed at the leaflet bases in *C.macrophylla* holotypes. Furthermore, significant variations in leaf length-width ratios, sizes, and positions were observed between different isotype specimens of *C.tangutorum* (as shown in Fig. 1).

**Figure 7.**
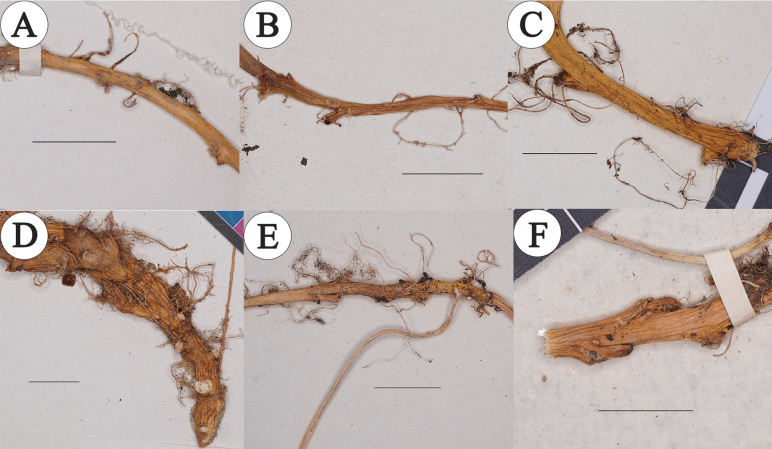
Rhizomes of *C.tangutorum* and *C.macrophylla* (**A, B***C.tangutorum***C–F***C.macrophylla*). Scale bar: 1 cm.

Upon examining the extant specimens of *C.macrophylla* and *C.tangutorum* in herbariums, we found that for both species, individuals with slender whip-like rhizomes have grooves on the rhizome surface but lack significant scales. However, some grayish-white triangular scales or leftover marks after their detachment were commonly present at the bases of leaf buds and branch buds (as shown in Fig. 7).

Considering that both *C.macrophylla* and *C.tangutorum* are perennial herbaceous plants with persistent rhizomes, these scaly appendages should be regarded as bud scales. In individuals with significantly fleshy rhizomes, these scales on the rhizome surface are less prominent. The presence or absence of scales on rhizomes, as emphasized in Schulz’s classification system, was considered an important criterion for subgroup classification. In Schulz’s system, a key feature of the *Cardamine* L. group is the prominent scales on rhizomes, whereas the macrophylla group lacks them. However, in The Families and Genera of Angiosperms in China it was suggested that the two groups in China should be merged, negating Schulz’s classification viewpoint (Wu et al. 2003). Examining the specimens, it is evident that the prominence of rhizome scales is affected by the plant’s age and the degree of rhizome fleshiness and does not serve as a basis for species differentiation. The quantitative taxonomy results indicate that none of the traditional differentiating traits effectively distinguish *C.macrophylla* from *C.tangutorum*. *C.macrophylla* shows significant morphological variability, within which *C.tangutorum* should be included.

Some species of *Cardamine* L., such as *C.yezoensis*, *C.pratensis*, etc., have variation in ploidy and relative DNA content within species (Marhold et al. 2010). The results of flow cytometry showed that the relative DNA content of *C.macrophylla* and *C.tangutorum* was stable within populations, but there was great variation among populations, showing polymorphism of ploidy and relative DNA content. However, the distribution range of DNA content of *C.macrophylla* and *C.tangutorum* were basically the same, which supported the combination of *C.macrophylla* and *C.tangutorum*.

Multiple populations of *C.macrophylla* and *C.tangutorum* were selected for the molecular phylogenetic study. The results indicate that both species share a high similarity in chloroplast genomes, with *C.tangutorum* nested within the monophyletic clade of *C.macrophylla*. This suggests close phylogenetic relationships at the molecular level, making it inappropriate to treat them as distinct species. *C.leucantha* and *C.fragarifolia*, with similar leaflet morphology to both *C.macrophylla* and *C.tangutorum*, all with long lanceolate leaflets and cuneate bases, are positioned within clade 3 in the molecular phylogenetic tree. This possibly suggests the single evolutionary origin of these morphological characters.

Combining molecular phylogenetic, cytological and morphological studies, it is concluded that the morphological range of *C.macrophylla* and *C.tangutorum* significantly overlaps, making them difficult to distinguish. Their molecular phylogenetic positions are nested within the same monophyletic group, rendering them indistinct. Considering the common species distributed, *C.macrophylla* and *C.tangutorum* should be treated as a single species, and *C.tangutorum* should be treated as a synonym of *C.macrophylla*.

### ﻿Taxonomic treatment

#### ﻿ Cardamine macrophylla

Taxon classificationPlantaeBrassicalesBrassicaceae

Willd., Sp. Pl. 3 (1): 484. 1800.

01A4DFF6-16FE-5C41-B715-2141E093B28A

=Cardaminemacrophyllavar.crenata Trautv., Trudy Imp. S.-Peterburgsk. Bot. Sada 5 (1): 18. 1877.
=Cardaminemacrophyllavar.dentariifolia Hook. f.& T. Anderson Fl. Brit. India [J. D. Hooker] 1 (1): 139 (1872). Type (designated by (Marhold et al. 2015)): India, Himal.Bor.Occ., *Thomson, T. s.n.*, (K000397478!)
=Cardaminemacrophyllavar.diplodonta T.Y. Cheo, Bull. Bot. Lab. N.-E. Forest. Inst., Harbin 6: 20. 1980.
=Cardaminefoliosa Wall.,[Numer. List: 4779. 1831. nom. nud.]
≡Cardaminemacrophyllavar.foliosa Hook. f.& T. Anderson Fl. Brit. India [J. D. Hooker] 1 (1): 139 (1872). Type(designated by (Marhold et al. 2015)): India, Kumaon, Wallich, Cat.Wall.4779 (K000247365!, P00747537!, B_10_0241370!, B_10_0241369!, GH00549127!).
=Cardaminemacrophyllavar.lobata Hook. f.& T. Anderson, Fl. Brit. India [J. D. Hooker] 1 (1): 139 (1872). Type(designated by (Marhold et al. 2015)): [INDIA] [Label 1]: “marshy meadows, Nira Zanskar, 12,900 ft, 2 July 1849 [?]”, [Label 2]: “Hab. Himal. Bor. Occ., W. Tibet, Regio Temp., Alt. 12,900 ft, T. T. [T. Thomson] s.n.” (K000397477).
=Cardaminemacrophyllavar.moupinensis Franch., Pl. David. 2: 18. 1888. Type(designated by (Marhold et al. 2015)): [CHINA], [Label 1 (handwritten)]: “Moupin, Thibet oriental, lieux frais en montagne, Avril 1869”, [Label 2 (printed)] “Chine (Thibet Oriental), Province de Moupin, 1870 [sic!], David s.n.” (P00747519, Isolectotype: P00747518).
=Cardaminemacrophyllavar.sikkimensis Hook. f.& T. Anderson Fl. Brit. India [J. D. Hooker] 1 (1): 139 (1872). Type (designed by (Marhold et al. 2015)): India, Sikkim Lachung, 03 September 1849, *Hooker, J.D. s.n.* (K000397479!, isolectotype: K000397480!, K000397481!, GH00549128!) =Cardaminepolyphylla D. Don not O. E. Schulz, Prodr. Fl. Nepal.: 201. 1825.
=Cardaminesachalinensis Miyabe & T. Miyake, Fl. Saghalin No. 58, t. 3, fig. 1–3, 1915.
=Cardaminesino-manshurica Kitag., Rep. Inst. Sci. Res. Manchoukuo 4: 111, 1940.
≡Dentariasino-manshurica Kitag.Rep. Inst. Sci. Res. Manchoukuo 4: 111, 1940.
=Cardamineurbaniana O.E. Schulz, Bot. Jahrb. Syst. 32 (2–3): 396. 1903.
Syntype: China, Sichuan, 1885–1888, *A. Henry 5635* (B 10 0241328!); China, Shaanxi, Huangcaoping County, 20 June 1894, *G. Giraldi 447* (B 10 0241329!)
=Dentariagmelinii Tausch, Flora 19 (2): 402, 1836.
=Dentariamacrophylla Bunge ex Maxim., Prim. Fl. Amur. 45, 1859.
=Dentariawallichii G. Don, Gen. Hist. 1: 172, 1831.
=Dentariawilldenowii Tausch, Flora 19 (2): 403, 1836.
=Cardaminetangutorum O.E. Schulz, Bot. Jahrb. Syst. 32 (2–3): 360. 1903.

##### Holotype.

Russia, Northern Sibiria, Mangezeya, at the mouth of Taz River (B-W11970-030!)

##### Syntype.

China, Gansu, Terra Tangurorum, *N. M. Przewalski No. 1872* (LE01014556!); China, Gansu, Terra Tangurorum, *N. M. Przewalski No. 1873* (K000697742!); China, Gansu, Terra Tangurorum, *N. M. Przewalski No. 1880* (LE01014557!); China, Gansu, orient, *G. N. Potanin 1885* (LE01014555!, LE01014558!); China, Sichuan, “Tsakulao”,1891, *A. v. Rosthorn 2583.*, (B 10 0241334!); China, Shaanxi, Baoji County, Mountain Miaowangshan, 1899, *J. Giraldi No. 3379*; China, Hebei, Mountain Xiaowutaishan, 1879, *O. V. Möllendorff s. n*.

##### Habitat.

Often in shady areas under forests, along ditches or in subalpine meadows.

##### Distribution.

Widely distributed in Siberia, Mongolia, Himalayas, North China, Northeast China, Central China, Hengduan Mountains.

##### Phenological period.

Flowering from May to July, fruit from June to September.

##### Description.

Perennial herbs, 30 cm–70 cm tall, up to 1 m. Rhizomes creeping, sometimes tuberous; basal leaves pinnate, pedicled on creeping rhizomes, terminal leaflet long ovate to lanceolate, margin toothed, lateral leaflet similar to terminal leaflet. Cauline leaves are similar to basal leaves but slightly smaller, mostly in the middle and upper parts of the plant. Flowers lilac to purplish red, calyx margins white membranous, petals with long claws; the seeds are oblong. This species showed high diversity in the size of leaflets and morphology of leaf margins.

##### Specimens examined.

• **Sichuan Province**: Yajiang County, Mountain Kazila, 08 September 2011, *He et al. SCU-11-360*, (KUN1235449!, KUN1235450!); Kangding County, *He et al. SCU-080340*, (KUN1235446!); • **Yunnan Province**: Shangri-La County, 24 July 2014, *Guo et al. 14CS9432*, (KUN1321027!); • **Heilongjiang Province**: Jinshantun County, Huilongwan National Forrest Park, *Hou et al. 389*, (QFNU0006577!, QFNU0006578!); • **Hebei**: Donggou Temple, 16 May 1951, *Wencai Wang 2122*, (PE 01004424!, PE 01004426!); Xiaowutai Mountain, Tielin Temple, 29 July 1906, Y. Yabe s.n., (NAS00326894!, NAS00326895!) • **Shanxi Province**: Mountain Guandishan, 23 June 1959, *Sai Ma 15030*, (WUK0321706!, WUK0324493!); Wutaishan Mountain, 17 July 1907, *Y. Yabe s. n.*, (NAS00326872); Lingchuan County, Fenghuang Valley, 14 April 2014, *Kong et al. k0087*, (SD00018202!, SD00016344!) • **Tibet Province**: Mangkang County, 318 Road, 20 July 2008, *Zhang et al. SunH-07ZX-0503* (KUN1300753!, KUN1300754!, KUN1300755!); Yadong County, Naiduila Mountain, 23 August 2013, *Y.S.Chen et al.13-1966*, (PE02000587!, PE02000588!); Dingqing County, 22 July 2016, *Shuai Li et al. 20167324*, (BNU0030842!); • **Gansu Province**: Tan County, Mountain Lougu, 15 June 1956, *Huanghe Exped 4911*, (WUK0085438!); Zhuoni County, Chebagou Valley, *Yin et al. LiuJQ-GN-2011-128*, (KUN1235443!); Maqu County, Xiuma, 03 September 2008, *Li et al. LiJ0061*, (KUN1235442!); Maqu County, Langmu Temple, 01 June 1999, *Bailong River Exped 1589*, (PE01556040!); Lianhua Mountain, 13 May 2002, *Xuegang Sun 2741*, (PE01998146!); Linxia County, Dalijia Mountain, 28 July 1988, *Ji Ma 88090*, (NAS00326893!, NAS00326896!, NAS00326897!); • **Beijing**: Baihua Mountain, August 1981, *Anonymous 81-0322*, (BJFC00019955!, BJFC00019954!, BJFC00019953!); Baihua Mountain, June 1990, *D. D. Lu et al. 272*, (BJFC00019961!, BJFC00019960!, BJFC00019959!, BJFC00019956!); Mentougou County, Lingshan Mountain, 01 May 2009, *G. M. Zhang 200923*, (BJFC00066271!, BJFC00066273, BJFC00066274!); • **Shaanxi**: Long County, 19 May 1983, Sujia River, J. X. Yang 4207, (WUK0438580!, WUK0438581!); • **Qinghai Province**: Ledu County, Qutan, 24 June 1970, *Benzhao Gou 7198*, (WUK0308377); Menyuan County, 26 July 2008, *Yuhu Wu LJQ-QLS-2008-0143*, (KUN1235444!, KUN1235445!); Maqin County, Dawu, 14 July 2014, *Xiaoyu Wu, Xiaolei Zhang s.n.*, QH2014006, (BNU0020082!); Huzhu County, Beishan Foresty Centre, 13 July 1982, B. *Z. Gou 25521*, (HNWP102005!); Guide County, Laji Mountain, 20 June 1992, *R. F. Huang 3710*, (HNWP169550!, HNWP169551!); • **Mongolia**: 17 August 1979, *Губанов 7845*, (MW0179858!, MW0179859!);17 August 1979, *Губанов 7830*, (MW0179855!, MW0179856!); 14 August 1979, *Губанов 7704*, (MW0179853!, MW0179854!); 9 August 1979, *Губанов 7610*,(MW0179857!, MW0179863!); • **Russia**, **Altai & Sayany Mountains**: 9 July 1984, Триль 4493, (MW0081495!); 27 July 1983, *Шауло 2019*, (MW0081478!); 17 July 1984, *Сонникова 1535*, (MW0081469!);18 June 1983, *Сонникова 1532*, (MW0081472!); 1 August 1982, *Сонникова 1529*, (MW0081468!); 8 August 1979, *Ухина 1518*, (MW0081474!); 25 June 1978, Ухина 1515, (MW0081471!); 13 June 1988, *Шауло 30*, (MW0081475!, MW0081494!); • **Russian Far East**: 16 August 1990, *Штрик 90-569*, (MW0081357!, MW0081364!); 23 August 1990, *Штрик 90-449*, (MW0081358!, MW0081372!); 8 August 1990, *Борисов 90-189*, (MW0081369!); 28 July 1988, *Кемниц 88-238*, (MW0081367!); 25 July 1998, *Кемниц 88-222*, (MW0081366!); • **Central Siberia**: 23 July 1977, *Куваев 85*,(MW0081457!) • **Baikal & Transbaikal region**: 9, August 1929, *Назаров 12.819*, (MW0081409!); 7 July 1929, *Назаров 12.118*, (MW0081404!) • **Kazakhstan**, **Western Altai Mountains**: 27, August, 1932, *Воронов 720*, (MW0081463!); 18 July 1930, *Смирнов 173*, (MW0081461!, MW0081462!).

## Supplementary Material

XML Treatment for Cardamine macrophylla

## ﻿Additional information

### Author ORCIDs

Jia-lu Li https://orcid.org/0009-0002-8147-5157

Yi He https://orcid.org/0000-0002-6925-7299

Quan-ru Liu https://orcid.org/0000-0003-4270-4746

### Data availability

All of the data that support the findings of this study are available in the main text.
